# Targeted agents in epithelial ovarian cancer: review on emerging therapies and future developments

**DOI:** 10.3332/ecancer.2016.626

**Published:** 2016-03-08

**Authors:** Rajitha Lokadasan, Francis V James, Geetha Narayanan, Pranab K Prabhakaran

**Affiliations:** 1 Department of Medical Oncology, Regional Cancer Centre, Thiruvananthapuram 695011, India; 2 Department of Radiotherapy, Regional Cancer Centre, Thiruvananthapuram 695011, India

**Keywords:** epithelial ovarian cancer, targeted agents, angiogenesis, bevacizumab, PARP inhibitors

## Abstract

Epithelial ovarian cancer (EOC) remains a clinical challenge and there is a need to optimise the currently available treatment and to urgently develop new therapeutic strategies. Recently, there has been improved understanding of the molecular characteristics and tumour microenvironment of ovarian cancers. This has facilitated the development of various targeted agents used concurrently with chemotherapy or as maintenance. Most of the studies have explored the tumour angiogenesis pathways. In phase-III trials, bevacizumab showed a statistically significant improvement in progression-free survival, although there was no improvement in overall survival in selected high-risk cases. Although several multi-targeted tyrosine kinase inhibitors were found to be useful, the toxicity and survival benefit has to be weighed. Poly ADP ribose polymerase (PARP) inhibitors have been another marvellous molecule found to be effective in breast cancer 1, early onset (BRCA)-positive ovarian cancers. Several newer molecules targeting Her 2, Wee tyrsine kinases, PIP3/AKT/mTR-signalling pathways, folate receptors are under development and may provide additional opportunities in the future. This article focuses on the targeted agents that have successfully paved the way in the management of epithelial ovarian cancer and the newer molecules that may offer therapeutic opportunities in the future.

## Introduction

### Epidemiology

Epithelial ovarian cancer (EOC) is the ninth most common malignancy in the world and the fifth most frequent cause of cancer death among women [1]. Ovarian cancer ranks the fifth for females in Europe and around 65,600 cases were diagnosed in 2012 [2]. In the United Kingdom, nearly, 4300 women died of ovarian cancer in 2012, which is around 12 every day. Despite all newer investigations and treatment modalities, even today a majority of females are diagnosed at an advanced stage (56% of cases diagnosed at stage III or stage IV) [2]. Inherited factors account for 5–15% of all ovarian cancers. Although there are several genes implicated in familial epithelial ovarian cancers, the majority are linked to BRCA 1 and 2 mutations [3]. Ovarian cancer risk is 65% higher in BRCA 1 mutations and 35% higher in BRCA 2 mutations [4]. Unfortunately, even today epithelial ovarian cancer remains one of the most deadly gynaecological malignancies in women.

### Clinical presentation and prognosis

EOC presents with vague symptoms, such as abdominal discomfort, abdominal bloating, bowel habit changes, fatigue, and early satiety. These subtle symptoms are often overlooked. Unfortunately, no screening programme has evolved. The five-year survival rates for patients diagnosed during 2002–2006 in the former Anglia Cancer Network were 90% for stage 1 and only 19% for stage 4 [5]. Most of the patients presented with stage-III disease and above.

### Treatment options

The present standard of care for all advanced EOC is optimal cytoreductive surgery and chemotherapy that includes platinum and taxanes.

In spite of optimal surgery and chemotherapy, approximately 80% of patients with epithelial ovarian cancer will relapse after first-line chemotherapy [6]. Recurrent ovarian cancer is divided into two groups for optimising treatment. The platinum-sensitive group includes those that recur after 6 months of completion of treatment. The platinum refractory and resistant group includes those that progress during or recur within 6 months of treatment. Primary chemotherapy resistance is observed in 15–20% of tumours.

Re-challenge with platinum-based treatment has been attempted in the platinum-sensitive group. A retrospective study at Memorial Sloan Kettering Cancer Centre (MSKCC) had shown a response rate based on the duration of platinum-free interval of 59% for more than 24 months, 33% for 13–24 months and 27% for 5–12 months [6]. The two challenges faced by patients with recurrent ovarian cancer are as ­follows: (1) Chemoresistance with repeated platinum administration; (2) Cumulative neurotoxicity if treatment is administered within one year completion of the treatment. Several salvage chemotherapy regimen has been used in platinum refractory cases – gemcitabine, ­liposomal doxorubicin, ifosfamide, topotecan, and docetaxel. The response rate is desperately low – 10% or less and the survival is extremely short. Hence, the intent of treatment for recurrent ovarian cancer is palliative and there is only very little prospect of cure.

With the recent development of targeted agents, it is hoped that the disease-free survival can be further extended with reduced toxicity. In this article, we review the various targeted agents approved in ovarian cancer at present and the new emerging therapies.

### Tumor microenvironment and targeted therapy

Carcinogenesis is a multistep process and tumour microenvironment an integral part. Strong evidence suggests that tumour cells depend on tumour microenvironment for carcinogenesis and growth. This knowledge has paved the way to target the vital molecules in tumour microenvironment to surpass carcinogenesis.

The two hallmarks of cancer that depend on the tumour microenvironment are as follows: (1) Angiogenesis; (2) Stromal invasion and metastasis. The tumour microenvironment consists of tumour cells, stromal cells, extracellular matrix molecules, and inflammatory cells. The interactions between these components allows for angiogenesis, stromal invasion, and metastasis to distant sites [7]. Cells should be within 100 μm reach of a capillary to receive sufficient oxygen and nutrients to sustain growth. Hence, for a tumour to increase beyond 1 mm in size angiogenesis should occur. Similarly, for metastasis to occur, the basement membrane needs to be invaded, which involves complex interactions between tumour cells and stroma. Understanding the molecular mechanisms of tumour growth and the interplay between stromal component, tumour cells, and cell-to-cell interactions is the major challenge.

## Targeting angiogenesis in ovarian cancer

Angiogenesis consists of a complex, dynamic, and highly regulated process driven by a delicate balance between pro- and anti-angiogenic factors in the cellular microenvironment [8]. The naturally occurring proangiogenic molecules are vascular endothelial growth factor (VEGF), fibroblast growth factor (FGF), and platelet-derived growth factor (PDGF). The antiangiogenic molecules are thrombospondin, endostatin, and angiostatin.

Vascular endothelial growth factor (VEGF) is a primary regulator of angiogenesis and vascular permeability. VEGF was first identified in 1999 by Ferrara *et al* [9]. The gene-encoding VEGF was mapped to chromosome 6p 21.3. There are seven family members of VEGF – VEGF A to E, placental growth factor (PGF) 1 and 2. VEGF A is the primary regulator of angiogenesis. VEGF A augments vascular permeability, mediates endothelial cell proliferation and migration, and allows cells to evade apoptosis. VEGF A has multiple isoforms of which VEGF 165 is commonly expressed in tumours.

**Figure 1.**
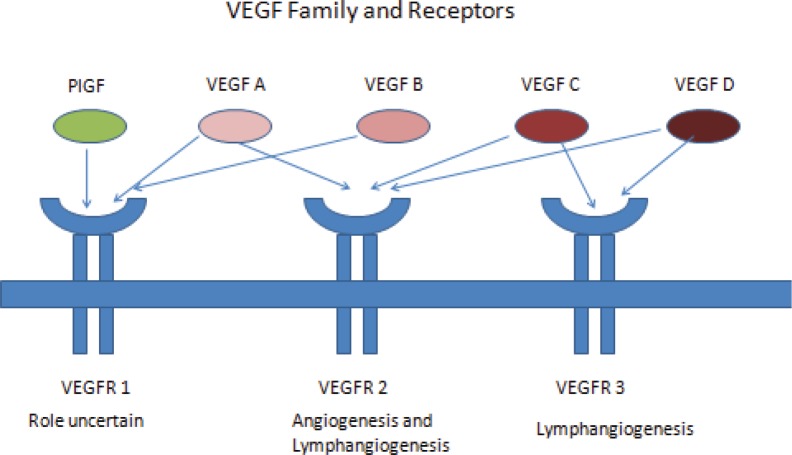
VEGF family and receptors.

VEGF signal through VEGF receptors (VEGFR). VEGFR are cell surface tyrosine kinase receptors and are of three types – VEGFR 1, 2, and 3. VEGF A, C, and D binds specifically to VEGFR. VEGFR 2 is the most important mediator of VEGF angiogenesis effects [9] (Figure 1).

Angiogenesis plays an integral role in the initiation and progression of ovarian carcinogenesis[10]. Retrospective studies have shown that an increased expression of VEGF and carriage of VEGF gene polymorphism are independent prognostic factors of ovarian cancer [11]. VEGF is expressed in higher levels in serous adenocarcinoma and clear cell tumours of the ovary. VEGF expression is demonstrated to be higher in advanced stage tumours as compared to those at an early stage. Experimental models have shown a positive correlation between ascites and VEGF level in ascitic fluid [12]. *In vitro* experiments by Schumacher *et al* have shown that mice injected with VEGF A excreting cells can transform normal ovaries to aggressive ascites producing ovarian cancers [13].

## Targeting VEGF ligand

### Bevacizumab

Bevacizumab is a recombinant humanised monoclonal IgG antibody that targets VEGF A. It binds to VEGF A, which neutralises it and thus suppresses tumour growth and dissemination. In addition, bevacizumab stabilises the tumour vasculature and decreases the permeability. This in turn can decrease the interstitial fluid pressure and increase tissue oxygenation. This can augment the effects of chemotherapy by improved delivery of chemotherapy to the tumour. This was initially demonstrated in animal models by Byrne *et al* in 2003 [14].

### Evolution of bevacizumab in ovarian cancer

Two phase-II trials had tested single agent. Bevacizumab in persistent or recurrent ovarian cancer – GOG 170D [15] by Burger *et al* and AVF 2949g by Cannistra *et al* [16]. Both these studies had evaluated the safety and efficacy of bevacizumab in patients with resistant ­epithelial ovarian cancer. Table 1 compares the two trials. The better PFS at 6 months and OS in GOG 170D were explained by the fact that only 36% of patients in the GOG 170D trial had platinum-resistant disease and none of them had received more than two chemotherapy regimens, while the AVF 2949g study was limited to patients with platinum-resistant disease (84%) and all patients had received two or more previous regimens. Table 2 compares the grade 3 and higher toxicities observed. More serious adverse effects occurred in the study by Cannistra *et al* [16]. The reasons for the differences in toxicity profile are not clear. Pre-existing small bowel obstruction and platinum-resistant disease were hypothesised by Cannistra *et al* to be associated with increased incidence of perforation while on bevacizumab therapy.

The encouraging results with bevacizumab in epithelial ovarian cancer paved the way for phase-II trials testing bevacizumab in combination with chemotherapy in heavily pre-treated recurrent ovarian carcinoma. Several hypotheses were proposed to highlight the benefit of combination. These included the fact that, in addition to the independent effects of both agents, bevacizumab can normalise the vasculature and improve the delivery of chemotherapy. The largest of these phase-II trials by Garcia *et al* had given bevacizumab 10 mg/kg intravenously every two weeks in combination with metronomic chemotherapy with low-dose cyclophosphamide in women with platinum-sensitive and platinum-resistant disease [17]. Similar retrospective studies in recurrent ovarian cancer had demonstrated response rates of 30–40% with an acceptable toxicity profile.

Many phase-II studies followed that evaluated bevacizumab in the first-line setting for advanced epithelial ovarian cancer. The taxotere, eloxatin, avastin in cancer of the ovary (TEACO study) is a phase-II study that evaluated bevacizumab in combination with oxaliplatin and docetaxel. The overall response rate was 58.6% with a one-year PFS of 65.7% (95% CI: 53.4%, 76.7%) [18]. A similar phase-II study by Micha *et al* tested bevacizumab in combination with carboplatin and paclitaxel in the adjuvant setting [19].

### Bevacizumab in the adjuvant setting and recurrent ovarian cancer

To date, there are four important phase-III trials that have addressed the efficacy of bevacizumab in combination with chemotherapy (Table 3). The first two trials tested bevacizumab in combination with carboplatin/paclitaxel in adjuvant setting (1) International Collaborative Ovarian Neoplasm (ICON 7) trial and (2) Gynaecologic Oncology Group (GOG) trial 218 [20, 21, 22]. The other two trials evaluated bevacizumab in recurrent cases of ovarian cancer: (3) Ovarian cancer study comparing efficacy and safety of chemotherapy and anti-angiogenic therapy in platinum-sensitive recurrent disease (OCEANS) and (4) Avastin use in platinum-resistant epithelial ovarian cancer (AURELIA) [23, 24, 25].

ICON-7 had enrolled 1528 patients with 70% of them having stage-IIIc or stage-IV ovarian cancer. About 30% of them had a high risk of progression defined as stage IIIc or IV with >1 cm residual disease following surgery. Carboplatin (AUC 5 or 6) and paclitaxel 175 mg/m^2^ was administered every three weeks for six cycles or this regimen plus 3-weekly bevacizumab (7.5 mg/kg) administered concurrently for 5 or 6 cycles and continued for 12 additional cycles or until disease progression [21, 22].

At a median follow-up of 36 months the bevacizumab arm showed a significant improvement in a median PFS of two months. This treatment effect was maximal at 12 months but diminished by 24 months. And a recent updated analysis did not show both a PFS and overall survival benefit in the bevacizumab arm [22]. However, an exploratory analysis of 502 patients who had disease with poor prognosis showed a significant difference in overall survival in women who received bevacizumab plus chemotherapy compared to those who received chemotherapy alone (restricted mean survival time 34.5 months [95% CI: 32.0–37.0] with standard chemotherapy versus 39.3 months [37.0–41.7] with bevacizumab; log-rank* p* = 0.03). Although in non-high-risk patients, the restricted mean survival time did not differ significantly between the two treatment groups (49.7 months [95% CI: 48.3–51.1]) in the standard chemotherapy group versus 48.4 months [47.0–49.9] in the bevacizumab group;* p* = 0.20).

The gynaecologic oncology group (GOG) protocol 218 was a three-arm placebo-controlled study. There was a total of 1873 women with advanced disease; 40% of patients had stage-III disease with residual disease of more than 1 cm and 26% who had stage-IV disease and had undergone debulking surgery were enrolled [20].

In the standard treatment, arm patients were given carboplatin (AUC 5 or 6) and paclitaxel 175 mg/m^2^ every three weeks for six cycles. In the bevacizumab throughout arm, bevacizumab was given with chemotherapy for 2–6 cycles and then continued every three weeks until a total of 22 cycles. In the bevacizumab initiation arm, bevacizumab was given with chemotherapy for 2–6 cycles and then continued with placebo in cycles 7–22. The dose of bevacizumab was given 15 mg/kg intravenous, which was double the dose given in ICON 7.

The improvement in median PFS was significant in the bevacizumab-throughout arm at a median follow-up of 17.3 months. There was no significant difference in overall survival between the three arms (Table 3).

Overall, the risk of toxicity with bevacizumab did not outweigh the clinical benefit. Bowel perforation, a concern in earlier studies was rare (1% of patients in ICON-7, 3% of patients in GOG 218). Other adverse events such as hypertension (grade 2 or higher in 18% of patients in ICON-7, 23% of patients in GOG 218), bleeding (mainly mucocutaneous), and thromboembolic events were slightly higher in GOG 218 than ICON-7 study.

The OCEANS trial was a randomised, multicentre, blinded, placebo-controlled phase-III trial. About 484 patients with platinum-sensitive recurrent ovarian cancer (recurrence 6 months after front-line platinum-based therapy) were enrolled [23]. Patients were randomly assigned to carboplatin plus gemcitabine combined with bevacizumab or placebo for six to 10 cycles. Bevacizumab or placebo was continued till disease progression. PFS for the bevacizumab arm was superior to that for the placebo arm with a four-month improvement (Table 3). In addition, there was a significant improvement in objective response rate (78.5% versus 57.4%;* p* < 0.0001) and duration of response (10.4 versus 7.4 months; HR, 0.534; 95%: CI: 0.408–0.698) with the addition of bevacizumab. There was no overall survival benefit for patients who received bevacizumab as compared to the placebo arm (33.6 months versus 32.9 months, respectively; hazard ratio = 0.95; log rank* p* = 0.65).

Grade 3 or higher hypertension (17.4% versus 1%) and proteinuria (8.5% versus 1%) occurred more frequently in the bevacizumab arm. Three patients in the bevacizumab arm had reversible posterior leukoencephalopathy syndrome and two patients had GI perforation.

The AURELIA trial was the first randomised phase-III trial evaluating bevacizumab in combination with chemotherapy in platinum-resistant ovarian cancer [24, 25]. The study enrolled 361 patients with ovarian cancer that progressed in less than 6 months after completion of platinum-based therapy. Pegylated liposomal doxorubicin 40 mg/m^2^ on day 1 every 4 weeks (*n* = 126), weekly paclitaxel at 80 mg/m^2^ on days 1, 8, 15, and 22 every four weeks (*n* = 115), or topotecan at 4 mg/m^2^ on days 1, 8, and 15 every four weeks or 1.25 mg/m^2^ on days 1 to 5 every three weeks (*n* = 120) were administered. Bevacizumab (10 mg/kg every two weeks or 15 mg/kg every three weeks) was given until progression, unacceptable toxicity, or consent withdrawal. There was a three months prolongation of PFS with the addition of bevacizumab. The OS trend was not significant (Table 3). ORR was higher in the bevacizumab arm 11.8% versus 27.3%, respectively (*p* = 0.001).

Bevacizumab-related adverse effects occurred in 57% of patients. Grade 3 adverse events included hypertension (7% versus 1%), proteinuria (2% versus 0%), GI perforation (2% versus 0%), thromboembolic events (5% versus 4%), fistula/abscess (1% versus 0%), and reversible posterior leukoencephalopathy syndrome (1% versus 0%).

AURELIA is the first of the bevacizumab combination studies to show an improvement in abdominal/GI symptom and other patient-reported outcomes. At week 8/9, a ≥ 15% improvement in abdominal/GI symptoms on the EORTC QLQ-OV28 was reported by 21.9% of patients in the bevacizumab/chemotherapy group versus 9.3% patients in the chemotherapy-alone group (difference = 12.7%,* p* 0.002). The GOG 218 and ICON7 studies either showed no improvement (GOG 218) or a slight worsening (ICON7) of patient-reported outcomes, and patient-reported outcomes were not examined in OCEANS(25).

Many questions remain to be answered regarding the optimal use of bevacizumab in patients with ovarian cancer. These include the optimal dose and duration of treatment. Although ICON-7 had used only half dose of bevacizumab in the adjuvant setting, which was attractive, GOG 218 supported prolonged use of bevacizumab. Another dilemma that remains unclear is whether bevacizumab maintenance needs to be extended till disease progression. Both the OCEANS and AURELIA trials in recurrent ovarian cancer had continued bevacizumab till disease progression.

Is the extent of benefit sufficient to justify the cost? The economic cost of bevacizumab in adjuvant and recurrent setting has not yet been addressed completely. Cohn *et al* evaluated the cost-effectiveness of bevacizumab in the GOG 218 study that included the actual and estimated costs of treatment and potential cost of complications. It was found that addition of bevacizumab to standard chemotherapy is not cost-effective [26].

Can biomarkers identify the groups more likely to benefit from bevacizumab? Although research into biomarkers predicting the response to bevacizumab therapy is underway, no single marker has been identified so far. Biomarkers, such as VEGF A, VEGFR 1, neuropilin, cell-free DNA in plasma, are all found to be promising [27, 28]. Gourley *et al* identified three major subgroups, two with angiogenic gene upregulation and one with angiogenic gene repression and immune gene upregulation. Addition of bevacizumab showed a trend towards improved PFS for the angiogenic gene upregulated subgroup [29]. A comprehensive gene analysis in the cancer genome atlas detected that high-grade serous ovarian cancer can be divided into the following four subtypes: differentiated, proliferative, mesenchymal, and immunoreactive subtypes and the response to bevacizumab validated in patients in the ICON 7 trial. A remarkable response was shown in the proliferative and mesenchymal subtypes, while the response was poor in other subtypes. This result highlights the need to identify robust predictive markers to assess response to bevacizumab that can guide the selection of patients in whom the drug is most beneficial [30].

All the positive phase-III trials of bevacizumab in ovarian cancer used PFS as the primary end point. It remains unclear if the improvement in progression free survival will translate to better quality of life or prolong survival. The lack of improvement in overall survival in GOG 218 may be due to the unblinding of patients at the progression-free survival analysis and crossover to bevacizumab following progression. This did not occur in ICON-7. And an exploratory analysis of high-risk patients in ICON-7 showed an overall survival advantage with bevacizumab. The use of bevacizumab in neoadjuvant, dose dense, and intraperitoneal therapy also needs to be further explored.

## Exploring beyond VEGF ligand in angiogenesis pathway

### VEGFR and other tyrosine kinase inhibitors

**Figure 2.**
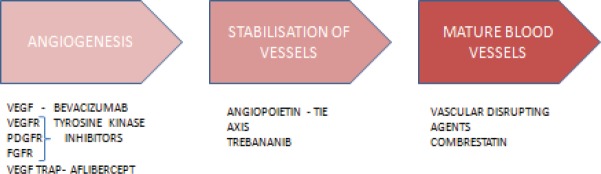
Sites of action of various antiangiogenesis agents.

Several VEGFR tyrosine kinase inhibitors are under evaluation either as monotherapy or as combination therapy in recurrent ovarian cancer. They act by inhibiting the activity of VEGFR and thereby block downstream signalling pathways. PDGF and FGF pathways have been implicated in resistance to anti-VEGF and VEGFR agents. Hence, counteracting these compensatory mechanisms by multitargeting other pro angiogenic pathways can improve the efficacy of anti VEGF therapies (Figure 2).

### Pazopanib

Pazopanib is an oral multikinase inhibitor of VEGFR, PDGFR, and c kit. A phase-II study of pazopanib monotherapy conducted in women who responded to standard therapy for ovarian cancer and had an increased CA 125 level was the first to demonstrate pazopanib activity in ovarian cancer with acceptable adverse effect profile [31].

The international Arbeitsgemeinschaft Gynaekologische OnkologieStudiengruppe Ovarialkarzinom trial 16 (AGOOVAR 16) was a phase-III randomised control trial (RCT) that evaluated the role of pazopanib in maintenance therapy of ovarian cancer [32]. About 940 patients who had not progressed after first-line platinum-based chemotherapy were randomised to receive either 800 mg of pazopanib once daily or placebo for up to 24 months. At a median follow-up of 24.3 months, PFS prolonged by 5.6 months and overall survival did not show benefit. However, the prolongation of PFS was at the expense of increased toxicity. Grade 3 or 4 adverse events included hypertension (30.8%), neutropenia (9.9%), liver-related toxicity (9.4%), diarrhoea (8.2%), fatigue (2.7%), thrombocytopenia (2.5%), and palmar-plantar erythrodysesthesia (1.9%). About 33% of patients in the pazopanib arm discontinued treatment and three patients had fatal adverse event – myocardial infarction, pneumonia, and posterior reversible encephalopathy syndrome. Moreover, it had excluded patients with bulky disease and also patients who progressed during chemotherapy.

Considering the increased toxicity and no survival benefit GSK had withdrawn the application to the European Union for approval of pazopanib in the maintenance therapy of ovarian cancer [33]. In an exploratory analysis, pazopanib appeared to have superior benefit in patients who were not of East Asian descent.

Randomisation in AGOOVAR 16 was done after completion of first-line treatment unlike GOG 218 which had included patients with stage-III disease and randomisation done at the time of diagnosis. Because of differences in trial designs, a head to head comparison between these trials cannot be made.

### Cediranib

Cediranib is an oral tyrosine kinase inhibitor of VEGFR and c-kit. The phase-II study conducted by Matulonis *et al* showed activity of cediranib in recurrent epithelial ovarian cancer [34]. Cediranib was administered at a dose of 45 mg daily and later the dose was reduced to 30 mg. Of the 47 patients enrolled, the clinical benefit rate was 30%. The median PFS was 5.2 months, and the median OS has not been reached. Grade 3 toxicities were reported in more than 20% of patients and included hypertension (46%), fatigue (24%), and diarrhoea (13%).

Cediranib was the first VEGFR TKI to show overall survival benefit when used as maintenance in recurrent ovarian cancer. This was demonstrated in the International Collaboration for Ovarian Neoplasia 6 (ICON6 trial) [35]. This was a three-arm, double-blind placebo-controlled phase-III trial that included 456 patients with platinum-sensitive recurrent ovarian cancer. They were randomised to one of the three cohorts: platinum-based chemotherapy with placebo maintenance, Concurrent chemotherapy with cediranib (20 mg per day) followed by placebo maintenance, Concurrent chemotherapy with cediranib followed by cediranib maintenance for 18 months. Cediranib maintenance arm showed improvement in progression-free survival and overall survival by 2 months (9.4 to 11.4 months, HR 0.68;* p* = 0.0022) and 2.7 months (17.6 t 20.3 months, HR 0.70;* p* = 0.0419), respectively. Although more adverse effects occurred in the cediranib arm this was found to be tolerable.

### Other multitargeted tyrosine kinase inhibitors

Several multitargeted small molecule tyrosine kinase inhibitors were tested in various trials as single agent or in combination in recurrent ovarian cancer (Table 4). The drugs such as sunitinib [36], sorafenib [37], cediranib [34], imatinib [38], and vandetanib [36] were widely tested. All these agents could demonstrate modest activity in recurrent ovarian cancer. However, these responses were attained at the cost of substantial toxicity.

### VEGF trap

This is an extracellular ligand-binding domain of VEGFR 1 and 2 fused to the constant region of IgG and act as a soluble decoy receptor modulating the availability of VEGF ligand. Aflibercept is a heterodimeric molecule consisting of domain of VEGFR 1 and VEGFR 2 with pmmunoglobin G Fc. The molecular weight of Aflibercept is lower than bevacizumab and has higher affinity for VEGF isoforms. This property generated interest in this new molecule.

Aflibercept was tested in a phase-II trial in patients with recurrent, platinum-resistant ovarian cancer who developed disease progression after receiving topotecan and/or pegylated liposomal doxorubicin [40]. Aflibercept 2 mg/kg or 4 mg/kg every 2 weeks was given until they developed disease progression or significant toxicity. The clinical benefit rate was only 12.3% and 11% in the 2 mg/kg and 4 mg/kg cohorts, respectively. This had not met the primary endpoint for response.

### Nintedanib

Nintedanib (BIBF 1120) is a triple angiokinase inhibitor that impedes angiogenesis at multiple levels by targeting VEGF, FGF, and PDGF. This was initially investigated for maintenance therapy in a phase-II trial with 83 patients having relapsed ovarian cancer who had completed chemotherapy [41]. Nintedanib was administered 250 mg twice per day for 36 weeks. Thirty-six-week PFS rates were 16.3% and 5.0% in the BIBF 1120 and placebo groups, respectively (hazard ratio, 0.65; 95% CI: 0.42–1.02;* p* = 0.06). This was offset by more toxicities in the form of diarrhoea, nausea or vomiting, grade 3 or 4 hepatotoxicity in patients on Nintedanib. The phase-III multicentric double-blind study AGO OVAR 12 that followed confirmed the significant prolongation of PFS in patients with advanced ovarian cancer [42]. Nintedanib in combination with carboplatin and paclitaxel was compared with placebo plus carboplatin and paclitaxel. (18.3 versus 16.6 months; HR, 0.84; 95% CI: 0.72–0.98;* p* = 0.0239)

## Inhibiting beyond VEGF pathway

### Angiopoietin

Angiopoietin 1 and 2 mediate separate actions upon binding with Tie2. Angpoietin1 impacts vessel quality, while Ang2 influences vessel quantity. Trebananib is a recombinant peptide Fc fusion protein (peptibody) that binds to angiopoietin-1 and angiopoietin-2 and inhibits their interaction with the Tie2 receptor. This in turn inhibits a signalling pathway that is normally involved in vascular growth, remodelling, and stabilisation.

TRINOVA-1 is a phase-III global, multicentre, randomised, double-blind, placebo-controlled study [43]. Over 900 women with recurrent partially platinum-sensitive or resistant epithelial ovarian cancer, primary peritoneal, or fallopian tube cancer who had received up to 3 prior regimens and had a platinum-free interval of less than 12 months were randomised to receive either 15 mg/kg of intravenous trebananib weekly plus 80 mg/m of intravenous paclitaxel weekly (three weeks on, one week off) or weekly intravenous placebo plus 80 mg/m^2^ intravenous paclitaxel weekly (three weeks on, one week off). A statistically significant improvement of 34% in the primary end point PFS (HR = 0.66; 95% CI: 0.57, 0.77, 0.001) was reported in the combination of paclitaxel and trebananib arm. The median OS showed a 1-month improvement which was not statistically significant.

The incidence of Grade 3+ adverse events was similar between treatment groups and the typical VEGFR inhibition-related adverse events were only slightly increased. TRINOVA-2 is a phase-III ongoing trial evaluating trebananib versus placebo in combination with pegylated liposomal doxorubicin in recurrent epithelial ovarian cancer. TRINOVA-3 is testing trebananib or placebo in combination with paclitaxel and carboplatin in the first-line treatment of epithelial ovarian cancer.

### Integrin receptors

Integrin receptors are involved in endothelial cell adhesion, migration, and proliferation. Integrin subunits α5β3 located on vascular endothelial cells and ovarian tumour cells has a prime role in tumour invasion and angiogenesis. Volociximab is an integrin inhibitor monoclonal antibody against chimeric. The α5β1 integrin that blocks binding of activated vascular endothelial cells to fibronectin is present in the extracellular matrix, thereby inducing apoptosis and inhibiting tumour growth. A phase-II three-arm adaptive randomisation trial studied the integrin inhibitor, volociximab (15 mg/kg q2wk or qwk) in combination with liposomal doxorubicin (PLD) compared with PLD alone. Disappointingly, the addition of volociximab was not superior to PLD alone [44, 45].

### Vascular disrupting agents

Vascular disrupting agents target mature established endothelial cells and pericytes. A phase-II trial tested combrestatin A in patients with relapsed ovarian cancer. Combrestatin was administered 63 mg/m^2^ minimum 18 h before paclitaxel 175 mg/m^2^ and carboplatin repeated every three weeks [46]. The addition of combrestatin to chemotherapy showed a high response rate of 13.5% with tolerable toxicity, and hence, the study was closed after 44 patients were recruited. A further randomised trial is required to test the drug efficacy.

### Vertical approach of VEGF inhibition

This implies targeting both the ligand and receptor. Several attempts have been carried out in ovarian cancer. A phase-I study combined sorafenib with bevacizumab. Although partial responses were seen in 6 of 13 patients (43%; response duration range, 4−22+ months), the regimen was not well tolerated [47]. An alternative approach that appears promising in early clinical trials is the combination of combretastatin and bevacizumab [48]. The combination can attenuate the revascularisation of the surviving tumour rim and thus potentiate the antitumour activity. Dual blockade of the VEGF and EGFR pathways bevacizumab in combination with erlotinib in recurrent ovarian cancer was not superior to bevacizumab alone and the incidence of grade-3 diarrhoea was higher than expected. Furthermore, fatal gastrointestinal perforations in 2 of the 13 recruited patients resulted in the closure of the study [49].

### PARP inhibitors

Poly ADP ribose polymerase inhibitors have opened a new paradigm in the treatment of ovarian cancer. The pharmacological strategy of targeting the DNA repair mechanism to selectively kill the tumour cells has been a new concept in cancer therapy. Excitingly, PARP inhibitors exploited the new concept of synthetic lethality [50].

Cellular DNA can undergo damage from endogenous and environmental factors. These DNA damages if not repaired in time will result in their collision with the replication fork resulting in the replication fork stalling or collapsing and the formation of double stranded breaks. The double-stranded breaks are in turn repaired by homologous recombination repair. The failure of one DNA damage response (DDR) pathway is compensated by the complementary mechanisms. Deregulation of DDR causes genomic instability and results in cancer development [51].

**Figure 3.**
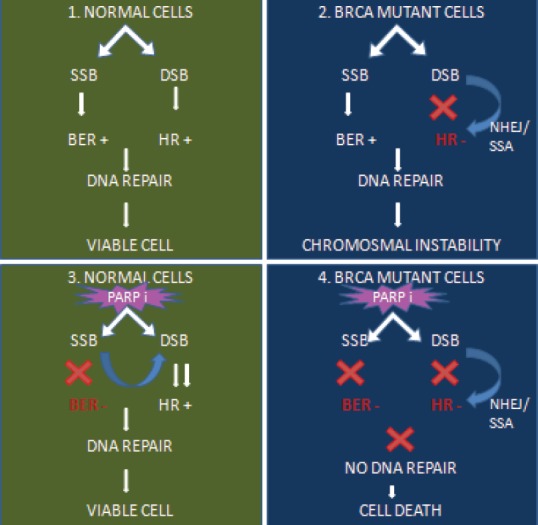
Concept of synthetic lethality.

Certain groups of cancer, such as breast and ovary, with BRCA 1 and 2 mutations have defective homologous recombination repair mechanisms. Hence, these cancer cells are more dependent on the complementary pathways, such as Base excision repair and single-strand base repair pathways for DNA repair and survival. Base excision repair and single-strand base repair pathways are PARP-dependent pathways. When PARP is inhibited the SSB will be unrepaired and tend to accumulate. The unrepaired SSB will cause replication stress and collapse replication forks resulting in replication-associated double-stranded breaks. Inactivated homologous recombination repair pathways cannot repair accurately the double-stranded breaks or gets repaired by error-prone mechanisms. Ultimately, this results in selective tumour cell death [50, 51] (Figure 3).

Pal T and group had conducted a population-based study of 232 women with EOC and found that up to 50% of them with high grade serous ovarian cancer were deficient in homologous recombination repair function due to germline mutation (15%) or somatically acquired (35%) [52]. No BRCA mutations were detected in borderline or invasive mucinous tumours. Dann R B *et al* demonstrated more frequent BRCA mutations in patients with platinum-sensitive epithelial ovarian cancers than in patients with platinum-resistant disease (38% versus 17%) [53].

Two works by Bryant *et al* and by Farmer *et al* had shown the spectacular sensitivity of BRCA 2-deficient cells to PARP inhibitors [54, 55]. Ma Cube *et al* had confirmed that deficiency of other proteins involved in HRR pathways RAD 51, RAD 54, DSS 1, RPA 1, NBS 1, ATR, ATM, CHK 1,2 FANCD2, FANCA, or FANCC induces sensitivity to PARP inhibitors [56]. This preclinical data suggested that PARP inhibitors may be useful not only in patients with germline BRCA mutations but also in a wide variety of tumours with dysfunction of genes involved in DNA damage response.

Fong *et al* in 2009 in a phase-I trial with olaparib first clinically corroborated the efficacy of PARP inhibitors in humans [57]. Subsequently, the international multicentre phase-II study by Audeh *et al* had enrolled women with BRCA 1/2 mutations and recurrent advanced ovarian cancer [58]. The first cohort received Olaparib 400 mg twice daily and second 100 mg twice daily. An overall response rate of 33% in the cohort assigned 400 mg twice daily and 13% in cohort assigned 100 mg twice daily was attained. The adverse events were well tolerated.

Olaparib was compared to chemotherapy (liposomal doxorubicin) in BRCA-mutated recurrent ovarian cancers that recurred within one year of platinum therapy [59]. Though olaparib 400 mg twice daily showed better response rates (31% and 18%), this was not statistically significant. There was no statistically significant difference in median PFS olaparib: 8.8 months; (95% CI: 5.4–9.2 months) and PLD 7.1 months (95% CI: 3.7–10.7 months). The results were consistent with previous studies and olaparib seemed promising in BRCA-mutant recurrent ovarian cancers.

Kaufman *et al* had tested olaparib monotherapy in patients with germline BRCA mutations. Among 193 patients with platinum-resistant ovarian cancer a significant tumour response rate of 31% (95% CI: 24.6–38.1) and stable disease (more than 8 weeks) in 40% patients (95% CI: 33.4–47.7) was demonstrated [60].

Based on all these data, an accelerated FDA approval for olaparib monotherapy 400 mg twice daily for patients with deleterious or suspected deleterious germline BRCA-mutant advanced ovarian cancer who had been treated with three or more prior lines of chemotherapy was granted in December 2014. Along with this, the FDA also approved the companion diagnostic test BRAC analysis CDx to identify patients with advanced ovarian cancer. This also ensures that eligible patients receive olaparib monotherapy.

The adverse effects associated with olaparib were nausea, vomiting, decreased appetite, anaemia, thrombocytopenia, arthralgia, and myalgia. Rare cases of acute myeloid leukaemia and myelodysplastic syndromes were reported in heavily treated patients [58, 60].

### Olaparib in maintenance

Dr. Jonanthan Ledermann in ASCO 2013 had presented the retrospective analysis of phase-II data of olaparib monotherpay as maintenance in advanced ovarian cancer patients with BRCA1/2 mutations [61, 62]. Patients with platinum-sensitive relapsed serous ovarian cancer who had already received two different platinum-based chemotherapies and had an objective and stable response with last treatment were randomised to receive as maintenance either olaparib 400 mg twice daily or placebo. Olaparib had shown 82% (*p* < 0.00001) reduction in risk of disease progression or death. This translated to median progression-free survival of 11.2 months in patients treated with olaparib compared to 4.3 months in patients treated with placebo. There was also a strong trend towards overall survival advantage, although this was not statistically significant (34.9 months versus 31.9 months) (*p* < 0.208). The overall survival benefit could not be shown probably because most patients received other post-progression therapies and lived for many more years.

The results support the hypothesis that platinum-sensitive recurrent serous ovarian cancers with a BRCA mutation have greatest likelihood of benefitting from olaparib treatment.

Based on these results, the European Medicines Agency approved olaparib as maintenance therapy in both germline BRCA-mutant and germline BRCA – wild-type recurrent ovarian cancer.

Two ongoing multicentred double-blinded randomised phase-III trials SOLO 1 and 2 are investigating the role of olaparib in the maintenance of high-grade ovarian cancer, fallopian tube and primary peritoneal cancer who have BRCAm tested by Myriad Integrated BRACAnalysis. SOLO1 includes patients with newly diagnosed, advanced ovarian cancer who have responded to first-line platinum therapy, whereas SOLO2 includes recurrent ovarian cancer patients who have completed ≥ 2 lines of platinum therapy.

### PARP inhibitors in combination with other agents

A lot of other studies of interest to clinical oncologists where olaparib is combined with other agents are coming up. An international, randomised phase-II trial combined olaparib with paclitaxel and carboplatin for 4 to 6 cycles followed by olaparib maintenance therapy in patients with recurrent platinum-sensitive serous ovarian cancer. This significantly increased progression-free survival by a median of 2.6 months versus no further therapy (*p* = 0.0012) [63].

PARP inhibitors are also being investigated in combination with other targeted agents. The combination of olaparib and cediranib in a phase-II study of the combination versus single-agent olaparib in patients with platinum-sensitive recurrent ovarian cancer prolonged progression-free survival, 17.7 months versus 9.0 months, over single-agent olaparib (HR = 0.42,* p* = 0.005). Patients with germline BRCA–wild-type ovarian cancer benefitted more. The reason postulated is that the hypoxia induced by cediranib potentiates the homologous recombination defects of the cell and leads to augmented PARP inhibitor activity [64]. All these have paved way to two phase-III studies: OVM1403 (for platinum-sensitive recurrent ovarian cancer) and OVM1405 (for platinum-resistant recurrent ovarian cancer).

### Newer PARP inhibitors

Racuparib is an oral potent inhibitor of PARP 1 and 2 and was recently granted breakthrough therapy designation by US FDA to Clovis Oncology. Rucaparib was approved as monotherapy treatment of advanced ovarian cancer in patients who have received at least two lines of prior platinum-containing therapy, with BRCA-mutated tumours, inclusive of both germline BRCA and somatic BRCA mutations based on two ongoing phase-II studies. The pivotal ARIEL 3 study (Assessment of Rucaparib in Ovarian Cancer Trial) compares the effects of rucaparib versus placebo.

Other PARP inhibitors, such as veliparib and niraparib, are also under investigation. Though PARP inhibitors are a promising class of drugs, more studies are needed to realise their full potential.

## Newer possible targets explored in ovarian cancer

### PI3K/AKT/mTOR-signalling pathway

The PI3K/PTEN/AKT pathway is a key-signalling pathway in the regulation of cell growth. Dysregulated signalling of this pathway can occur with activating mutations of PI3K-related genes, amplification of Akt signalling or inactivating mutations of PTEN. This in turn promotes growth factor-independent growth and increase cell invasion and metastasis. It was found that Akt and PI3KCA mutations were mutually exclusive [65].

PI3K/Akt/mTOR-signalling pathway is frequently deregulated in ovarian cancer. This abnormal signal transduction is found to be histology specific. In high-grade serous ovarian cancer, PIK3CA mutations and PTEN loss occur in 20% and 5%, respectively. However, 35% of clear cell ovarian cancers have PIK3CA mutations. Endometroid ovarian cancers document 20% PIK3CA mutations and 20% PTEN loss of function mutations [66, 67]. Patients with PIK3CA mutations treated with PI3K/AKT/mTOR inhibitors demonstrated a higher response rate than patients without mutations [68, 69]. Currently, a phase-II trial is investigating the efficacy of temsirolimus, carboplatin, and paclitaxel as first-line therapy in patients with newly diagnosed stage-III–IV clear-cell ovarian cancer.

### Human epidermal growth factor 2

Encouraging results with trastuzumab in breast cancer have evoked considerable interest in ovarian cancer as well. Although 70% of patients with ovarian cancer show low-level expression of Her2, focal high-level expression is seen only in 20%. The prognostic significance of Her2 over expression in ovarian cancer is rare and has no prognostic value. Hence, it can be understood that the biology of Her2 over expression in ovarian cancer is different from in breast cancer. Single-agent trastuzumab in advanced ovarian cancer has shown a poor response rate [70], while pertuzumab in combination with gemcitabine in platinum-resistant ovarian cancer showed a response rate of 13.8% as compared to 4.6% in patients receiving only gemcitabine [71, 72]. An ongoing trial is investigating the efficacy of pertuzumab in combination with conventional chemotherapy topotecan, paclitaxel, or gemcitabine. At the same time, Her3 has been attributed to poor prognosis and resistance to chemotherapy in ovarian cancer. Hence, new strategies targeting Her3 or survivin can improve the efficacy of chemotherapy.

### WEE1 tyrosine kinase inhibitors

The WEE1 kinase is an important regulator of G2 cell-cycle check point that prevents entry into mitosis until DNA replication has been completed [73]. Cancer cells due to mutations of G1 regulators like p53 depends on G2 arrest for DNA repair. Wee1 inhibition can thus increase antitumour activity. Certain ongoing clinical trials are looking into the efficacy of Wee1 inhibitors in combination with chemotherapy in platinum-resistant p53-mutated ovarian cancers.

### Folate antagonist and folate receptor alpha antagonist

Yet another potential therapeutic target in ovarian cancer is folate and its membrane-bound receptor. Cancer cells depend strongly on folate metabolism. Recently, the anti-tumour potency of thymidylate synthase inhibitors, such as pemetrexed, has been identified [74]. A phase-II study combined pemetrexed with bevacizumab in 34 patients with resistant epithelial ovarian cancer and attained a attractive median PFS of 7.9 months and OS of 25.7 months [75]. About 80% of ovarian cancers express a folate receptor that is absent from normal cells [76]. Hence, monoclonal antibodies to folate receptor farletuzumab have gained interest in trials [77]. However, initial phase-III trials with farletuzumab in ovarian cancer did not meet the primary end point. A phase-II PRECEDENT study used vintafolide (EC145), a folate conjugated with desacetyl vinblastine hydrazine in patients with recurrent platinum resistant ovarian cancer who had undergone less than three prior cytotoxic regimens [78]. Patients were randomised to receive either pegylated liposomal doxorubicin as single-agent therapy or in combination with vintafolide. Median PFS was 5.0 and 2.7 months for the vintafolide + PLD and PLD alone arms, respectively. Subsequently, a phase-III ‘Study for women with platinum-resistant ovarian cancer evaluating EC145 in combination with DoxilR (PROCEED)’ was initiated and is currently recruiting patients.

### Targeted therapy in low-grade EOC

Type-I EOC includes low-grade serous, mucinous, and clear-cell carcinoma. They are biologically distinct entities with indolent clinical behaviour and resistant to chemotherapy. In contrast to type-II high-grade EOC, they are less frequently associated with p53 mutations and are more genetically stable. However, driver mutations in mitogen-activated protein kinases (MAPK) pathway have been identified in low-grade serous EOC. In a phase-II study, 52 women with recurrent low-grade serous EOC were given selumitinib 50 mg twice daily orally till progression [79]. About 15% of patients demonstrated objective response rate showing activity of selumitinib. Similarly, Her2 amplification was identified in 20% of mucinous EOC and was found to be mutually exclusive with KRAS mutations [80]. There are only a few studies that have demonstrated the activity of trastuzumab in her2 amplified EC [70]. Ovarian clear-cell carcinoma of the ovary is yet another emerging zone of genetic and phenotypic heterogeneity. ARID1A AT-rich interactive domain 1A is one such key somatic mutation and warrants further exploration [81]. The important pathways identified are PI3PK/Akt/mtor, loss of PTEN expression are other potential therapeutic targets [82]. Several ongoing clinical trials are investigating the role of these novel molecular targets in the treatment of low-grade EOC.

### Immune therapies in ovarian cancer

Immune therapy in ovarian cancer is in an early phase of development restricted only to clinical trials. Novel and promising molecules have shown clinical activity and may play a central role in the coming years. Immune check point inhibitors are under investigation in ovarian cancer. Anti-CTLA 4 (cytotoxic T-lymphocyte-associated protein 4) monoclonal antibody ipilimumab has shown anti-tumour effect in stage-IV ovarian cancer [83]. Another upcoming molecule is anti-PD1 (programmed cell death 1) antibody that accomplish activity via effector T-lymphocytic cell. Nivolumab is a fully humanised IgG4 that blocks the engagement of PD-1 by PD-1 ligands. This was one of the first trials that demonstrated the effect of nivolumab in patients with platinum refractory ovarian cancer and was presented in ASC 2014 [84]. Trials on vaccines and adoptive T-cell therapy approaches are going on. Abagovomab is a murine anti-idiotypic antibody against the antigen CA-125 and has shown responses against ovarian cancer [85]. Anther promising area is the antibody–drug conjugates, for example, anti-MUC 16 antibody–drug conjugates or the anti-NaPi2b antibody–drug conjugates has demonstrated clinical response. Ovarian cancer is known to have a complex immune microenvironment but only further evaluation can elucidate how much immunotherapy can contribute to treatment.

## Conclusion

Ovarian cancer still remains one of the most challenging cancers to treat. Although much development has been made recently in the treatment of ovarian cancer the prognosis is still dismal. Over the last decade, ovarian cancer prognosis has improved very modestly. These advances are most likely from surgery and platinum-based therapy. Despite the strong rationale of clinical trials considering genetic targets, most of the studies have shown only modest benefit in terms of progression-free survival. Other than two very recent approvals – for olaparib and bevacizumab, there have not been any new therapies approved for ovarian cancer in almost 10 years.

Although ovarian cancer is a targetable tumour, they are biologically distinct and highly heterogeneous both at diagnosis and later at recurrence. The unique biology of ovarian cancer with tumour heterogeneity and genetic instability may contribute to resistance to single-targeted therapy. We believe that ovarian cancer has short cuts and secret passages that are not yet fully discovered. The more we try to explore and understand the molecular mechanisms and tumour biology the more complicated it becomes. Hence multitargeted therapies with minimal toxicities are the need of the hour. Discovery of the complex connections and dynamics of these pathways may help us develop novel therapeutic targets to fight this aggressive tumour.

One of the first promising targeted agents in the management of ovarian cancer is bevacizumab. With the available data, the European Medicines Agency has approved the use of bevacizumab for use in first line setting in FIGO stage IIIB disease, recurrent ovarian cancers both platinum sensitive and platinum refractory. Despite the positive results in clinical trials many questions remain unaddressed. With the available treatment options, the question of whether bevacizumab should be used as first line or in a recurrent setting needs to be answered. Oza *et al* in ICON 7 trial noticed the overall survival benefit with bevacizumab in a first-line setting in a subset of high-risk patients with inoperable or suboptimally cytoreduced stage-III or stage-IV disease. However, caution should be taken while interpreting the results from a subset analysis where the consistency of the patient population is not ensured. GOG 218 looked into the role of bevacizumab in the front-line setting in females with advanced ovarian cancer (stage III with residual disease more than 1 cm and stage IV after debulking surgery) and could demonstrate comparable advantage in progression-free survival. At the same time, Oza *et al* could identify three subgroups – clear cell, low-grade serous, and high-risk low-stage cancer – less likely to benefit from bevacizumab. All these findings raise the question as to whether patients with high-risk disease benefit from frontline bevacizumab. In the recurrent ovarian setting (platinum sensitive and platinum refractory), although trials showed remarkable improvement in progression-free survival none could demonstrate overall survival benefit. The improvement in quality of life in the Aurelia trial was much appreciated as the treatment options in platinum-resistant recurrent ovarian cancers is limited and patients often present with refractory abdominal symptoms.

There are several ongoing trials evaluating the optimal timing of bevacizumab, duration of treatment, benefit in continuing beyond progression and re-challenging bevacizumabin ovarian cancers. There is ongoing debate related to the cost benefit ratio of bevacizumab. And given the high cost of bevacizumab, patients who receive bevacizumab should be carefully selected. Further research validating biological predictive markers to assess response to bevacizumab is the need of the hour.

The second recently approved drug is olaparib. FDA has approved olaparib as monotherapy for women who have germline BRCA mutations and have been treated with three or more lines of previous chemotherapy as detected by the companion diagnostic test BRAC Analysis CDx . While European Medicines Agency approved olaparib as maintenance therapy in BRCA-mutant (germline and/or somatic) relapsed platinum-sensitive ovarian cancer who are in complete or partial response to platinum-based therapy.

Strategies combining parp inhibitors with antiangiogenic agents are under investigation. Research is ongoing for the potential enhancement of sensitivity to PARP inhibitors by increasing homologous recombination defects through changes in oxygenation caused by anti-angiogenic agents. For example, cediranib in ICON 6 trial and olaparib as maintenance in recurrent ovarian setting has shown impressive benefit. Further evaluation is needed before approval of these drugs in combination for ovarian carcinoma.

Another area of research that has been expanding fast is in targeted therapy of low-grade ovarian cancers. Similarly immunotherapies – including vaccines, checkpoint inhibitors, or adoptive approaches – though in early stage is promising.

To conclude, at present PARP inhibitors and antiangiogenic treatment has changed the standard treatment in ovarian carcinoma. Undoubtedly, future research for better understanding of molecular mechanism and targeting combination therapies to overcome resistance may help conquer this disease with minimal toxicity. More tailored treatments based on molecular characteristics are expected in the years to come.

## Figures and Tables

**Table 1.** Bevacizumab monotherapy in recurrent ovarian cancer — a comparison between GOG 170D [15] and AVF 2949 g [16].

	GOG 170D [15]	Cannistra *et al* [16]
No of patients enrolled	62	44 (terminated prematurely)
PFI < 6 months	36%	84%
No of Previous regimens (1/2/3/4)	34/66/0/0	0/52/48/0
GOG/ ECOG PS (0/1/2)	73/27/0	59/41/0
Response rates	21%	16%
PFS > 6 months	40%	28%
OS in months	17	10.7

PFI: platinum-free interval; PFS: progression-free survival; OS: overall survival.

**Table 2.** Compares grade 3 and higher toxicities between GOG 170D [15] and AVF 2949 g [16].

Grade-3 toxicity and above	GOG 170D [15]	Cannistra *et al* [16]
Gastrointestinal perforation	0	3(11%)^*^
Arterial thromboembolism	0	3(8%)^*^
Hypertension grade 3	6(10%)	6(14%)^*^
CNS	0	1
Proteinuria	1	0
Grade-3 toxicity and above	None	41%

* Febrile neutropenia.

**Table 3.** Phase-III data for bevacizumab in adjuvant setting and recurrent ovarian cancer.

TRIAL	ARMS	Setting	PFS (HR, *p* value)	OS (HR, *p* value)
ICON 7 [21] *N* = 1528	CP versus CP + B → B (7.5 mg/kg) 12 cycles	Adjuvant	At 36 months follow-up 17.4 versus 19.8 [0.87; 0.004]	Restricted mean survival time 44.6 versus 44.5 *p* = 0.85
GOG 218 [20] *N* = 1873	CP versus CP + B versus CP + B → B (15 mg/kg) 22 cycles	Adjuvant	10.3 versus 11.2 versus 14.1 [0.908; 0.16] [0.717; < 0.001]	39.3 versus 38.7 versus 39.7 [1.036; 0.76] [0.915; 0.45]
OCEANS [23] *n* = 484	CG + placebo versus CG + B	Recurrent ovarian cancer	8.4 versus 12.4 [0.484; < 0.0001]	33.7c versus 33.4c [0.960; 0.736]
AURELIA [24] *n* = 361	CT (PLD, P or Top) versus CT + Bev	Recurrent ovarian cancer	3.4 versus 6.7 [0.48; < 0.001]	13.3 versus 16.6 [0.85; 0.174]

CP – carboplatin and paclitaxel, B – Bevacizumab, CG – carboplatin and gemcitabine,

CT – chemotherapy, PLD – liposomal doxorubicin, P – pacliltaxel, top – topotecan,

PFS – progression-free survival, OS – overall survival, HR – hazard ratio.

**Table 4.** Tyrosine kinase inhibitors in recurrent ovarian cancer.

TKI	Site of action	RR	Median PFS (m)	Median OS (m)	Grade 3/4 toxicities
Sunitinib Biaji *et al* [36]	VEGFR, PDGFR	3.3%	4.1	–	Fatigue, hypertension, HFS, neutropenia
Sorafenib Mateo *et al* [37]	VEGFR, PDGFR, Flt3, c kit, Raf	3.4%	2.1	16.33	Rash, HFS, metabolic, cardiac and pulmonary toxicity.
Cediranib Matulonis *et al* [34]	VEGFR, c kit	17%	5.2	Not reached^*^	CNS haemorrhage, hypertriglyceridaemia and elevated creatinine.
Vandetanib^**^ Harter P *et al* [39]	VEGFR, EGFR, RET	10%	6.7	11.1	Elevated liver enzymes, neutropenia, pulmonary embolism, mucositis.

TKI: tyrosine kinase inhibitors; RR: response rates; PFS: progression-free survival; m: Months; OS: overall survival; HFS: hand foot syndrome.

* Mean OS was 16.3 months.

** Vandetanib was combined with liposomal doxorubicin 50 mg/m^2^ Q 28-day cycle.
